# Different protein-protein interface patterns predicted by different machine learning methods

**DOI:** 10.1038/s41598-017-16397-z

**Published:** 2017-11-22

**Authors:** Wei Wang, Yongxiao Yang, Jianxin Yin, Xinqi Gong

**Affiliations:** 1 Center for Applied Statistics and School of Statistics, Renmin University of China, Beijing, 100872 China; 2 Mathematics Intelligence Application LAB, Institute for Mathematical Sciences, Renmin University of China, Beijing, 100872 China

## Abstract

Different types of protein-protein interactions make different protein-protein interface patterns. Different machine learning methods are suitable to deal with different types of data. Then, is it the same situation that different interface patterns are preferred for prediction by different machine learning methods? Here, four different machine learning methods were employed to predict protein-protein interface residue pairs on different interface patterns. The performances of the methods for different types of proteins are different, which suggest that different machine learning methods tend to predict different protein-protein interface patterns. We made use of ANOVA and variable selection to prove our result. Our proposed methods taking advantages of different single methods also got a good prediction result compared to single methods. In addition to the prediction of protein-protein interactions, this idea can be extended to other research areas such as protein structure prediction and design.

## Introduction

Protein-protein interactions are of great importance in living organisms. Many biological processes are accomplished with the association and dissociation of different proteins. With the advancement of biotechnologies, the relevant data regarding protein-protein interactions become more and more sufficient^1^. Protein-protein interactions can be categorized into different types according to their biological functions or other features^2^. Different interface patterns made by these protein-protein interactions could be characterized by different types of data such as mRNA expression, essentiality, localization and functional annotation which imply different interaction mechanisms.

The formation pattern of protein-protein complex interface is crucial for protein-protein interactions and recognitions. The importance and difficulty of theoretically understanding and computationally predicting the interface pattern have attracted many scientists trying to make the underlying mechanism clear^3–5^. Although some individual and simply combined prediction methods have been proposed, usually different prediction methods give out different results^6–8^. What makes a surface residue of a protein monomer become an interface one when the monomer binds to the partner? Is it possible to predict all the specific kind of interface pattern uniformly and correctly using specific methods? These are our interests.

It is well known that different machine learning methods are suitable to deal with different kinds of statistics problems. For example, classification problem can be used to treat with Support Vector Machine^9^, Random forest^10^ and so on while clustering problem can be used treat with K-means^11^, DBSCAN^12^ and so on. When faced with a specific problem, one always prefers a commonly used or newly developed algorithm, but it is difficult to prove that the selected algorithm is the best one compared to the other unused algorithms. Usually, one compares several algorithms on different datasets to prove that one of those algorithms is the most effective under certain criteria but there are few literatures analyze why different algorithms make the difference. And there is no golden criterion that tells researchers what method is applicable for the new coming data. Different machine learning methods on microarray gene expression data and IP traffic flow^13,14^ were compared systematically to construct a better framework for these data, which inspires us to consider the differences between different methods when analyzing protein dataset.

**Figure 1:**
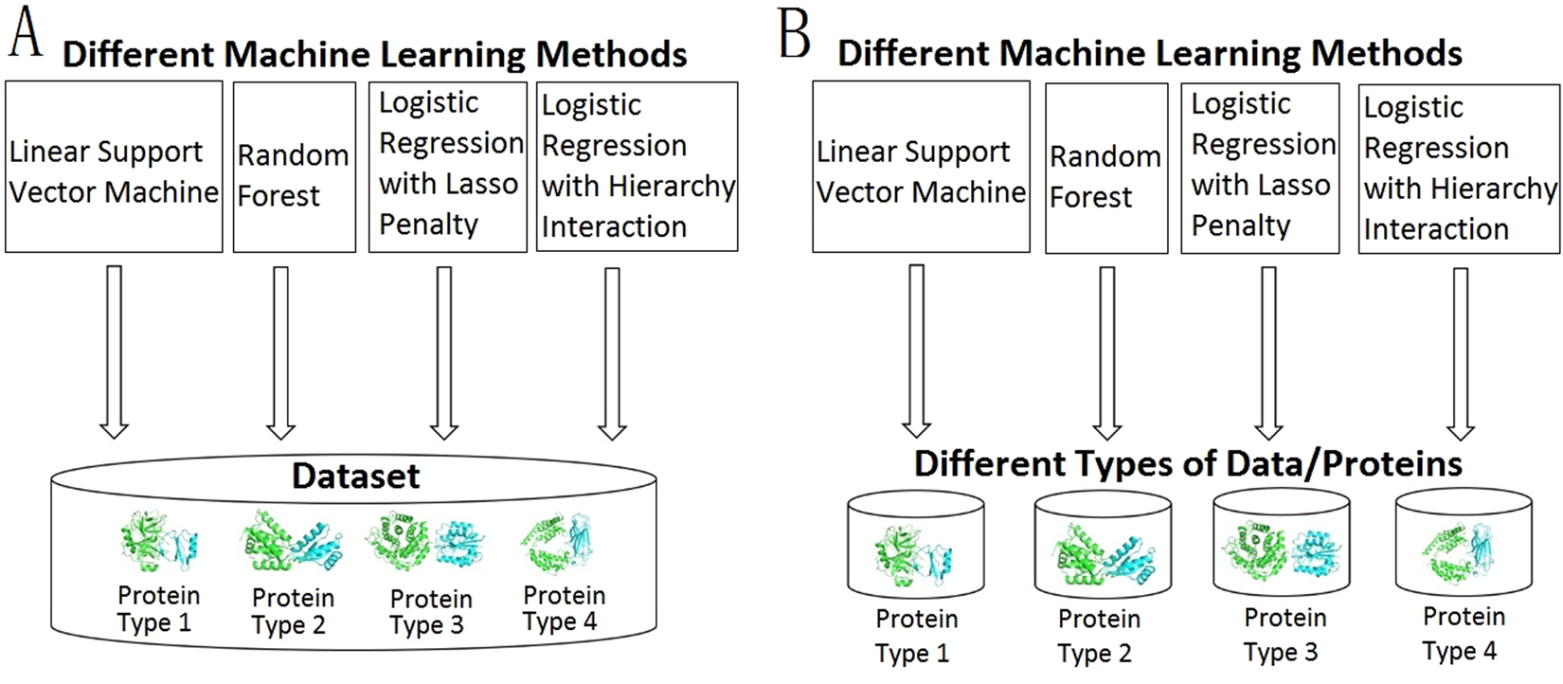
Sketch map of the two ways to find rules in a dataset. (**A**) Traditional way to find a general rule. Different machine learning methods are used to find a general rule in the whole dataset. (**B**) Alternative way to find some simple rules decomposed from a general role. Different machine learning methods prefer to find the different rules implied in different types of data or proteins.

In fact, many machine learning methods have been used to predict protein interfaces in the past years^15–19^. But the performances of different machine learning methods for different types of proteins have not been investigated. In general, the performances of different methods will be compared in the whole dataset to find a general rule (Fig. 1A). It doesn’t work when the rule is extremely complicated. The complicated rule can be decomposed into some simple ones. These simple rules are implied in different types of proteins which are suitable to be tackled by different machine learning methods (Fig. 1B).

Here, we used four machine learning models including linear support vector machine, random forest, logistic regression with lasso penalty and logistic regression with hierarchy interaction to train effective models for predicting interface residue pairs of different protein-protein complexes. The complexes were described and distinguished using several attributes containing their nature properties and indexes we constructed. Then, we evaluated the preferences of different machine learning methods on predicting different classes of dimers.

## Methods

### Models

In this paper, we used linear SVM, random forest, logistic regression with lasso penalty and logistic regression with hierarchy interaction to forecast the interacting pair-residues. By comparing the results of these four methods, we want to learn how different methods treat protein-protein data and to find their applicability. Afterwards, we briefly introduced the basic ideas and advantages of the four methods.

Given a training dataset of n points $({{\boldsymbol{x}}}_{i},{y}_{i}),i=1,\mathrm{..}.,n,{y}_{i}\in \{-1,1\}$. If there exists a vector ***w*** and a scalar *b* which make the following inequalities hold:

1 $$\begin{array}{c}{\boldsymbol{w}}{{\boldsymbol{x}}}_{i}+b\ge 1\,for\,{y}_{i}=1\\ {\boldsymbol{w}}{{\boldsymbol{x}}}_{i}+b\le {-}1\,for\,{y}_{i}=-1,\end{array}$$

we can define that the training data are linearly separable. Linear SVM^3^ tries to find optimal linear hyperplane

2 $${\boldsymbol{w}}{{\boldsymbol{x}}}_{i}+b=0,$$

which separates two classes of data and the distance between two classes is maximal. SVM is based on the principle of structural risk minimization and the final decision function of SVM is determined by important support vectors. It means that SVM can alleviates problems of over-fitting and is stable for new data^16^. SVM is convex optimization problem so it avoids local minimal problem. Besides, by defining a suitable kernel, SVM is able to generate nonlinear decision boundaries and classify data without obvious vector space representation^20^. SVM is widely used in computational biology^20^ due to its large-margin nature and good accuracy over previous unseen examples^18^. In the field of protein-protein interactions, linear SVM^16^, SVM with Gaussian kernel^21^ and SVM with pairwise kernels^18^ were used to predict interacting residues and got good performance.

Random forest^10^ uses bagging algorithm to combine a multitude of decision trees and output the mode class of these trees. Random forest has the strengths of decision trees and are more robust. Besides, importance of variables can be obtained in a natural way through random forest. Random forest can perform well with imbalance data and high dimensions because it selects features and training examples randomly for each tree and outputs importance of features^19^. However, when the noise in data is large, it faces the problems of over-fitting. Benefits from the easy adjustment, high robustness and suitability for high dimension data and imbalanced data, random forest has often been used for protein-protein interactions. Chen and Liu^22^ and Zhu-Hong You *et al*.^23^ inferred protein interactions using random forest with novel feature representation while Mile šikić *et al*.^19^ used parallel random forest.

Given a training dataset of n points $({{\boldsymbol{x}}}_{i},{y}_{i}),i=1,\mathrm{..}.,n,{y}_{i}\in \{0,1\}$. Logistic model^24^ is as follows:

3 $$P{(}{{y}}_{i}=1{)}=\frac{\exp ({\beta }_{0}+{{\boldsymbol{x}}}_{i}^{{\rm{T}}}{\boldsymbol{\beta }}{)}}{{1}+\exp ({\beta }_{0}+{{\boldsymbol{x}}}_{i}^{{\rm{T}}}{\boldsymbol{\beta }}{)}},$$

and the log-likelihood is

4 $$L{(}{\boldsymbol{y}}{|}{\beta }_{0},{\boldsymbol{\beta }}{)}\,=\sum _{i=1}^{n}[{y}_{i}({\beta }_{0}+{{\boldsymbol{x}}}_{i}^{{\rm{T}}}{\boldsymbol{\beta }}{)}-\,{log}{(}{1}+{\exp }{(}{\beta }_{0}+{{\boldsymbol{x}}}_{i}^{{\rm{T}}}{\boldsymbol{\beta }}{)}].$$

The loss function is defined as ${-}L{(}{\boldsymbol{y}}{|}{\beta }_{0},{\boldsymbol{\beta }}{)}\,$. By adding a penalty, we can get a logistic model with variable selection function whose coefficients are obtained by solving

$$\mathop{{\rm{argmin}}}\limits_{{\beta }_{0},{\boldsymbol{\beta }}}-\frac{1}{n}L{(}{\boldsymbol{y}}{|}{\beta }_{0},{\boldsymbol{\beta }}{)}\,+\lambda \sum |\beta |.$$

Logistic model applies maximum likelihood estimation and outputs probability between 0–1 so it is easy to operate to real data and can be well interpreted. When we assume that the features are independent in a classification problem, it is a good choice to adopt logistic model. Several logistic models were employed to predict protein-protein interactions such as kernel-based logistic regression models^25^ and logistic regression with lasso penalty^26^.

Logistic regression with hierarchy interaction^27^ learns pairwise interactions with strong hierarchy which means that an interaction is estimated nonzero only if its main effects are estimated nonzero. The model is

$${\text{logit}}{(}P{(}{{y}}_{i}=1{)}{)}=\mu {+}\,\sum _{i=1}^{p}{x}_{i}{\theta }_{i}+\sum _{i < j}{x}_{i:j}{\theta }_{i:j}{,}$$

and

$${\text{logit}}{(}t{)}=\,{ln}\,\frac{t}{1-t},$$

where ${x}_{i:j}={x}_{i}\times {x}_{j}$ and ${\theta }_{i:j}$ means the interaction effect between *x*_*i*_ and *x*_*j*_.

The log-likelihood of logistic regression with hierarchy interaction is

$$L{(}{\boldsymbol{y}}{|}\mu ,{\boldsymbol{\theta }}{)}\,=\sum _{i=1}^{n}[{y}_{i}(\mu +\sum _{i=1}^{p}{x}_{i}{\theta }_{i}+\sum _{i < j}{x}_{i:j}{\theta }_{i:j}{)}{-}\,{log}{(}1+{\exp }{(}\mu +\sum _{i=1}^{p}{x}_{i}{\theta }_{i}+\sum _{i < j}{x}_{i:j}{\theta }_{i:j}{)}].\,$$

By adding overlapped group-lasso penalty into the log-likelihood, we can fit logistic model with hierarchy interaction while selecting variables.

Logistic regression with hierarchy interaction consider pairwise interaction effects that satisfied strong hierarchy so it mines more information of data and perform better compared to logistic regression when the model is identifiable. In other words, logistic regression with hierarchy interaction considers the dependence of features and expands the feature space. As a result of researches such like SVM considers pairwise kernels and random forest considers feature representation, it is reasonable to consider the interaction effects between features especially between receptor protein and ligand protein. So comparing logistic regression with hierarchy interaction to logistic regression with lasso penalty helps to conclude the independence of features and find more important features.

In protein-protein interactions prediction, there doesn’t exist a best method suitable for all data. To compare the performance of different methods in protein-protein interactions prediction, Nan Lin *et al*.^2^ compared random forest, logistic regression and the Bayesian network method and Yanjun Qi *et al*.^28^ compared decision tree, logistic regression, Naïve Bayesian, SVM, random forest and kernel random forest. Without prior information, we excluded Bayesian method. Instead, we considered logistic regression with hierarchy interaction to supplement the representation of features. Therefore, we finally chose linear SVM, random forest, logistic regression with lasso penalty and logistic regression with hierarchy interaction as the methods of study.

### Algorithms

**Figure 2:**
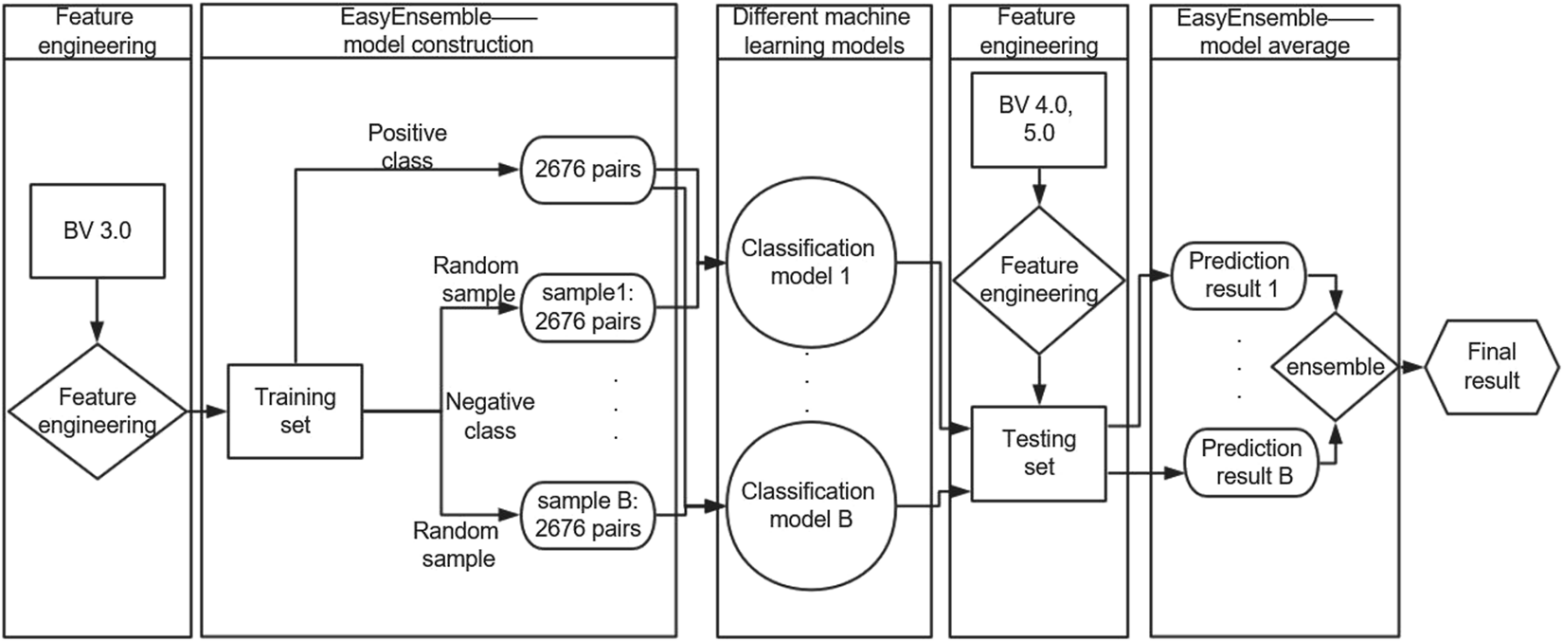
Algorithm flow chart. Training set and testing set were constructed by expending the features of BV 3.0, BV 4.0 and BV 5.0. Sample subset of negative class was combined with positive class to generate a balance training sample dataset. And the size of Sample subset of negative class was set to be the same with that of positive class that was 2676. The balance training sample can be trained by one of four classification models shown before and the tuning process was described in Supplementary. Then the model was used to predict testing set and the estimation probabilities of all models on testing set were collected. We compared different ensemble methods using mean, median and weighted mean of the estimation probabilities of all models on testing set. And the results were shown in Fig. 3. The weights were chosen to be the numbers of correct predicted dimers on training set. And a dimer was regarded to be correct if the top 20 residue pairs chosen by final result contained at least 1 interactive residue pair.

The data used in this paper are protein-protein docking benchmark version 5.0^29^, which contained 67 unbound state dimers. The benchmark version 5.0 (BV 5.0) was updated from benchmark version 3.0 (BV 3.0) and benchmark version 4.0 (BV 3.0). We chose the unbound dimers in BV 3.0 as our training set which contained 34 dimers. BV 4.0 and BV 5.0 were used to test the effects of different methods. Number of surface residue pairs of BV 3.0 was 1306311 and only 2676 residue pairs interacted. The other details of data were given in “data” section of Supplementary. Each residue pair contained 18 features of 9 receptor features and 9 ligand features. These features described geometric information and hydrophilic information of residue pairs, which was in “variables” section of Supplementary in detail. We faced imbalanced problem and small amount of features when dealing with this data. To improve the performances of the methods, EasyEnsemble^30,31^ and feature engineering were adopted. The whole algorithms was shown in Fig. 2. The details about the tuning process of model were in the “tuning” section of Supplementary.

EasyEnsemble algorithm was developed to deal with class imbalance problem. Interface residue pair prediction is such a problem because the percentages of true interface residue pairs among all the possible residue pairs made by the surface residues from different monomers are very low (Table S1). Class imbalance is induced by the great difference between the numbers of the samples belonging to different classes. If it is difficult to discriminate one class from the other classes according to the descriptors, class imbalance will make the problem more difficult. If the descriptors are powerful, class imbalance is not a problem. Although the algorithm is just a palliative, it is effective to improve the performances of the methods. This algorithm is also helpful in variables selection according to stability selection method^32^. The main idea of stability selection is to record selected variables in each model and select high frequency variables among these models.

Besides the problem of imbalance, we also faced a few variables of big sample. As we known in deep learning process, initial inputs are transferred by some functions to large number of nodes which contains the information of inputs. The amount of nodes are often times of the amount of inputs. Although the nodes are hard to be explained but after recoding they can make sense in prediction. Inspired by the feature engineering ideal of deep learning, we have expanded our variables by adding difference, quadratic and divided terms without losing interpretability. Like the machine learning methods, feature engineering is also used to explore the best performances of the original features as the amount of our original features is only 18 which is very small compared to the sample size. By feature engineering, that is expanding our original features, features are more variously represented and easier to mine their own information. The linear or nonlinear discriminants made by feature engineering can not only be directly used to predict interface residue pairs but also be regarded as new features and the input of the machine learning methods. In this paper, feature engineering algorithm expended 18 original variables standardized by BV 3.0 to 243 variables including 18 original standardized variables, 9 differences items between receptor and ligand residues, 27 quadratic terms of variables of receptor, ligand residues and their differences items, 18 × 17/2 divided terms of original variables of receptor, ligand residues and 9 × 8/2 divided terms of differences items.

## Results

### Improvement of the performances of the methods without distinction of protein types

**Figure 3:**
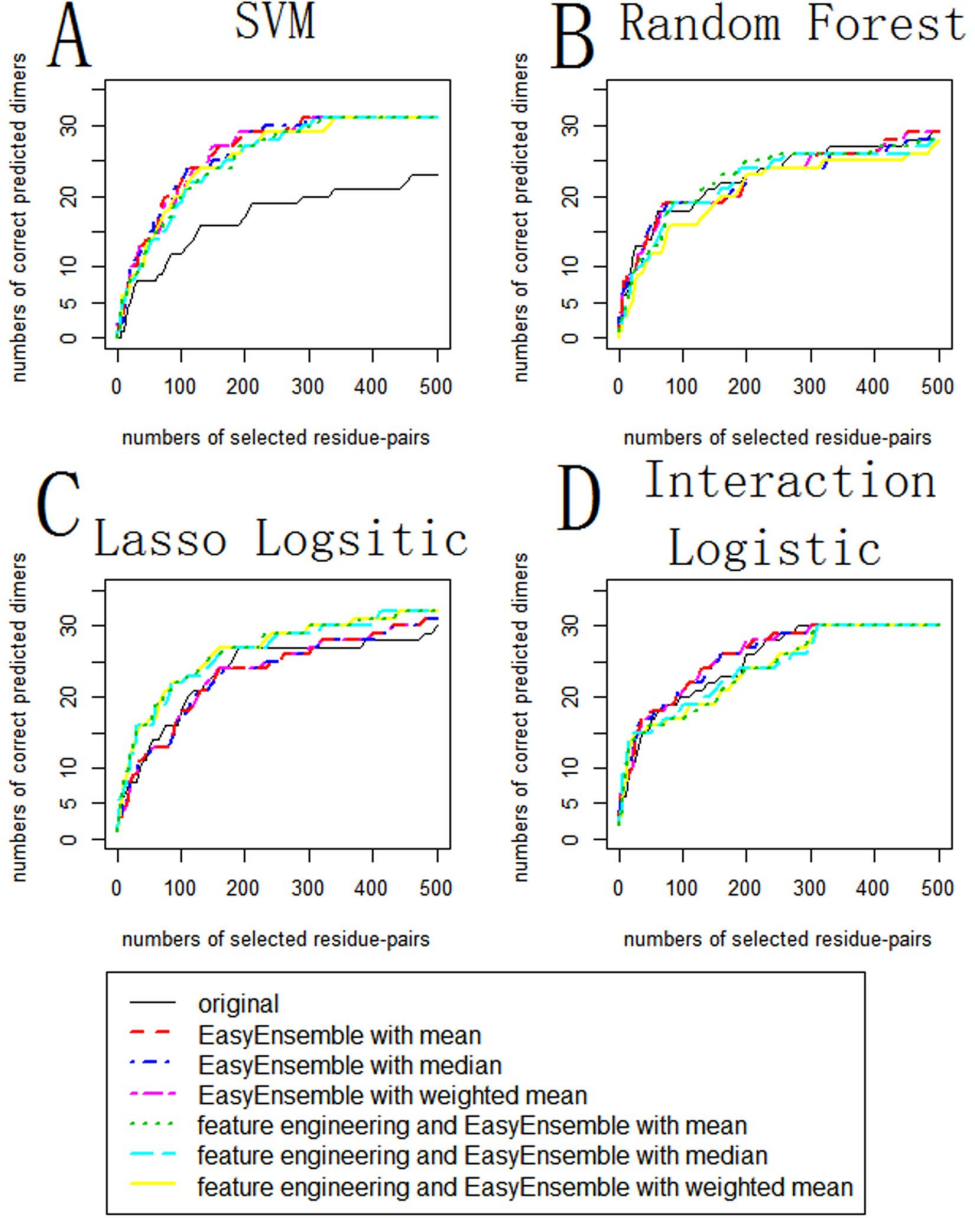
Effects of three different algorithms in four methods. (**A**,**B**,**C**,**D)** each presents the results of SVM, random forest, logistic regression with lasso penalty, logistic regression with hierarchy interaction. The abscissa means numbers of residue pairs chosen to be interacting residue pairs in a dimer and the ordinate means numbers of correct predicted dimers as long as there is one truly interacting residue pair chosen correctly. In the legend, “original” means algorithm without EasyEnsemble and feature engineering, “EasyEnsemble” means algorithm that only using EasyEnsemble without feature engineering, “feature engineering and EasyEnsemble” represents the results obtained by both EasyEnsemble and feature engineering. In addition, “mean”, “median” and “weighted mean” indicated three ensemble methods that were used in both “EasyEnsemble” and “feature engineering and EasyEnsemble”.

In order to test the effects of algorithms, BV 4.0 and BV 5.0 were predicted in different algorithms. We compared the results of three different treatments and three different ensemble methods on four different classification models. One of three treatments used original data without EasyEnsemble and feature engineering. One only used EasyEnsemble without feature engineering. And the other used both EasyEnsemble and feature engineering as shown in Fig. 2. And we used mean, median and weighted mean in both algorithm with only EasyEnsemble and algorithm with EasyEnsemble and feature engineering. The final results were shown in Fig. 3.

First, we compared the effects of three ensemble methods. As we could see from Fig. 3, except the result of weighted mean in the feature engineering and EasyEnsemble algorithm in random forest was pooler than the results of mean and median in the feature engineering and EasyEnsemble algorithm in random forest, results of three ensemble methods using mean, median and weighted mean of the estimation probabilities in each algorithm were similar. Especially in logistic regression with lasso and logistic regression with hierarchy interaction, we could see that three ensemble methods were almost identical. And the poor result of weighted mean in the feature engineering and EasyEnsemble algorithm in random forest might be blame to the over-fitting problem of random forest on training set. Because the results of median and weighted mean didn’t show significant excellent than that of mean, so we still used the most simple and interpretable ensemble method which used the mean of the estimation probabilities in the following analysis.

Second, from Fig. 3 we found that EasyEnsemble increased the prediction effect while feature engineering didn’t help in improving forecast effect for SVM. For random forest, three treatment performed similarly as we know that random forest already contains ensemble process. For logistic regression with lasso penalty, the result of EasyEnsemble algorithm without feature engineering seemed similar to the result of original data. While the result of EasyEnsemble algorithm and feature engineering was much better than other results of treatments. For logistic regression with hierarchy interaction, the result of EasyEnsemble algorithm and feature engineering was the best when the number of selected residue pairs was small in each dimer. When the number of selected residue pairs became large, the result of EasyEnsemble algorithm without feature engineering was better than the results of the other two treatment.

Combined with the properties of four classification methods, we had the following results. First, it told us that ordinary linear SVM doesn’t deal with the problem of imbalance and is sensitive to variables. It’s better to choose significant variables for linear SVM. Second, as random forest is an ensemble method of trees, it already treated the problem of variable selection and imbalance so EasyEnsemble and feature engineering didn’t help much for its prediction. Third, for logistic regression with lasso penalty, EasyEnsemble and feature engineering helped a lot for prediction, which suggested that there are indeed many hidden information in 18 variables that is not found by logistic regression and needed to be added in by feature engineering or other algorithms. At last, EasyEnsemble improved the prediction effect for logistic regression with hierarchy interaction compared to original treatment, which suggested us that logistic regression with hierarchy interaction also need to deal with the problem of imbalance. EasyEnsemble and feature engineering improved the prediction effect when the number of selected residue pairs was small but its effect didn’t hold when the number of selected residue pairs increased, which told us that EasyEnsemble and feature engineering do find more information but need to be more robust.

### Comparison of the best performances of the methods without distinction of protein types

We showed the forecast situations of top 20 residue pairs of four models using different treatments on BV 4.0 and BV 5.0 in Table 1. It’s not unexpected that the outcomes of two dataset are inconsistent because we have showed in data and variables section (Supplement) that three dataset may have different distribution and the percentage of interacting residue pairs differs up to a factor of two between BV 4.0 and BV 5.0. In BV 4.0, we saw that logistic regression with hierarchy interaction perform best but in BV 5.0, logistic regression with lasso penalty had highest prediction accuracy. Especially we found that the accuracies of SVM and logistic regression with lasso have little difference between the prediction of BV 4.0 and BV 5.0 while the accuracies of random forest and logistic regression with hierarchy interaction reduce a lot from the prediction of BV 4.0 to BV 5.0. It told us that SVM and logistic regression with lasso may find more general patterns of protein-protein data while random forest and logistic regression with hierarchy interaction may find more details of data and present the characteristics of over fitting.

**Table 1:** Prediction results of top 20 residue pairs on BV 4.0 and BV5.0 set^[a]^. NSRP: Number of Surface Residue Pairs, obtained by multiplying the number of residues on the surface of receptor and ligand^[b]^; NIRP: Number of Interacting Residue Pairs, given by^23^. The result of SVM was got only using EasyEnsemble. The result of random forest was got without EasyEnsemble and feature engineering. The results of logistic regression with lasso penalty, logistic regression with hierarchy interaction were obtained using both EasyEnsemble and feature engineering.

Dimer BV 4.0	NSRP^[a]^	NIRP^[b]^	SVM	RF	Logistics	Interaction logistic
1CLV	12928	91	1	1	2	2
1FFW	7316	41	2	2	1	5
1GL1	7548	71	1	2	2	2
1H9D	14796	99	0	0	0	0
1MQ8	25725	59	0	0	0	0
1OC0	12456	46	0	1	1	2
1OYV	25536	105	0	2	0	1
1R6Q	10611	62	2	0	0	1
1SYX	7080	46	0	0	0	0
1US7	22184	44	0	0	0	0
1ZM4	144948	65	0	0	1	0
2A5T	65260	73	0	0	0	0
2ABZ	16226	69	0	1	2	1
2G77	44688	94	0	1	0	0
2I9B	30360	107	0	0	0	1
2J0T	18600	71	1	0	0	0
2O3B	26875	69	1	0	0	1
2OUL	22568	93	0	1	2	1
2VDB	30672	75	0	0	0	0
4CPA	10526	56	0	0	0	1
**BV 5.0**	**NSRP**	**NIRP**	**SVM**	**RF**	**Logistics**	**Interaction logistic**
2GAF	117448	129	0	0	1	0
2GTP	35910	59	0	0	1	1
2YVJ	36531	61	2	0	2	1
3A4S	11476	38	0	2	1	0
3AAD	37548	59	0	0	0	0
BAAD	37548	58	0	0	0	0
3FN1	16490	79	0	1	0	0
3K75	32109	54	0	1	0	0
3S9D	31408	69	0	0	0	0
3VLB	78764	96	1	0	1	1
4H03	138852	59	1	0	0	0
4IZ7	28014	44	0	0	0	0
4M76	39772	43	0	0	0	0
Accuracy			0.018	0.023	0.026	0.032
Correct dimers			9	11	12	14

**Figure 4:**
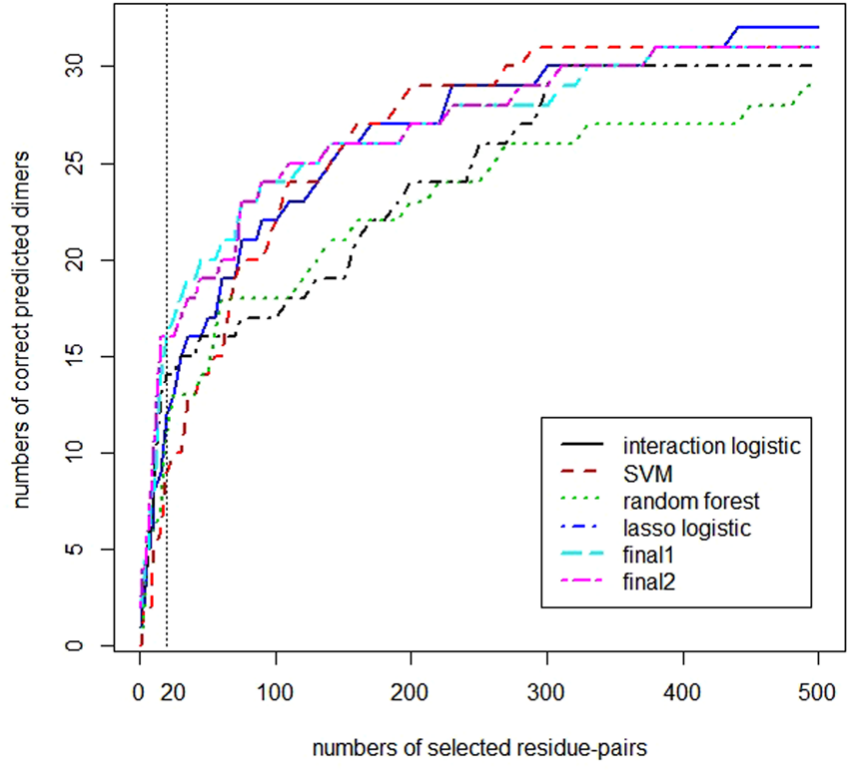
Prediction results of six methods including two proposed methods by us and four single methods mentioned before. The abscissa means the number of residue pairs chosen to be interacting residue pairs in a dimer and the ordinate means the number of correct predicted dimers as long as there is one truly interacting residue pair chosen correctly. The results of logistic regression with lasso penalty and logistic regression with hierarchy interaction were obtained by both EasyEnsemble and feature engineering. We got the result of SVM by only using EasyEnsemble. The outcome of random forest was predicted without EasyEnsemble and feature engineering. The method used to get “final 1” and “final 2” was the method we proposed, in which “final 1” presented using random forest and “final 2” indicated using logistic regression with hierarchy interaction when NSRP was less than 20000. The vertical dotted line pointed out the difference of six methods choosing the number of selected residue-pairs in each dimer to be 20.

Let us observe the prediction results of four methods on BV 4.0 and BV 5.0 in more detail through Fig. 4. We could see that the logistic regression with hierarchy interaction performs not well when abscissa is not too small nor too large both on BV 4.0 and BV 5.0. Other three methods didn’t have this phenomenon in prediction process. And when the number of selected residue pairs was smaller than 30, the performance of logistic regression with hierarchy interaction was the best of four methods. Logistic regression with lasso penalty was always a good choice no matter how the number of selected residue pairs change. Its accuracy was only lower than that of logistic regression with hierarchy interaction when the number of selected residue pairs was less than 30 and lower than that of SVM when selecting more than 120 residue pairs in prediction each dimer. SVM performed the worst when the number of selected residue pairs was less than 60 while when the number of selected residue pairs was larger than 120, SVM became the most accurate method. The performance of random forest was always not satisfying except when the number of selected residue pairs was between 55 and 70.

The machine learning methods with the best performances changed with the increasing of the number of selected residue pairs in a dimer from logistic regression with hierarchy interaction to logistic regression with lasso penalty to SVM. There was no machine learning method that was superior to the other methods all the time. One reason may be that there doesn’t exist a universal discriminant to recognize interface residue pairs on different protein-protein interfaces.

### Relationship between different machine learning methods and different proteins

Table 1 showed some similarity between four methods. For example, in Table 1 the first three dimers were correctly predicted by all four methods and some dimers were not being correctly predicted by all the four methods. We thought this phenomenon may be caused by the collective effect of attributes of different dimers and properties of different classification methods. Firstly, it was a simple idea that the size of dimer may have an effect so we used NSRP (Number of Surface Residue Pairs) to represent size. We calculated the percentage of interaction residue pairs in each dimer recorded as P to characterize the activity of a dimer. Besides, we constructed an easy Euclidean distance recorded Distance to indicate the dispersion of interacting residue pairs and non-interacting residue pairs. To calculate Distance, first standardize 18 original features of residue pairs in individual dimers, then calculate the Euclidean distance of the mean of features of interacting residue pairs and non-interacting residue pairs. In addition, we used complex category labels^29^ to describe dimers.

To show the relationship between prediction results and several dimer attributes, we formed Table 2 by finding the rank of first true positive interacting residue pair of each dimer on BV 4.0 and BV 5.0 using four methods with different algorithms. From Table 2, we saw that dimers such as 1CLV, 1FFW and 1GL1 that had small NSRP and large P were well predicted by all methods while dimers like 2A5T, 3AAD and 4M76 that had large NSRP and small P were not well predicted by all methods. So we could approximately conclude that larger P, smaller NSRP help the prediction of interacting residue pairs. But when NSRP were especially large such like 1ZM4 and 2GAF, logistic regression with lasso penalty had a good prediction. And the category of dimers also mattered in prediction. If the dimer belonged to EI, it seemed to be easy to be predict correctly. If the dimer belonged to OX, it seemed to be hard to be predict correctly even it had small NSRP and large P such like 1H9D and 1SYX.

**Table 2:** Rank of the first detected true positive residue pair in each dimer of four methods. This table shows the rank of first true positive interacting residue pair of each dimer in four methods^[a]^. The percentage of interaction pairs^[b]^; The Euclidean distance between interacting residue pairs and non- interacting residue pairs^[c]^; Complex category labels: antibody-antigen (A); enzyme-inhibitor (EI); enzyme-substrate (ES); enzyme complex with a regulatory or accessory chain (ER); others, G-protein containing (OG); others, receptor containing (OR); others, miscellaneous (OX)^23^.

Dimer BV4.0	NSRP	NIRP	SVM	RF	Logistics	Interaction logistic	P^[a]^	Distance^[b]^	category labels^[c]^	category number
1CLV	12928	91	11	7	4	12	0.0070	1.52	EI	1
1FFW	7316	41	9	4	10	2	0.0056	2.04	OX	8
1GL1	7548	71	10	4	16	1	0.0094	1.25	EI	1
1H9D	14796	99	66	24	149	75	0.0067	1.23	OX	8
1MQ8	25725	59	55	787	230	242	0.0023	1.52	OX	8
1OC0	12456	46	194	16	18	10	0.0037	1.70	ER	3
1OYV	25536	105	31	5	27	13	0.0041	1.34	EI	1
1R6Q	10611	62	16	329	104	5	0.0058	1.42	ER	3
1SYX	7080	46	108	120	222	103	0.0065	2.29	OX	8
1US7	22184	44	96	488	74	247	0.0020	2.09	ER	3
1ZM4	144948	65	157	1147	2	122	0.00045	2.76	ES	2
2A5T	65260	73	62	1107	60	155	0.0011	1.79	OX	8
2ABZ	16226	69	110	5	5	9	0.0043	1.30	EI	1
2G77	44688	94	283	19	33	151	0.0021	1.33	OG	6
2I9B	30360	107	140	56	24	12	0.0035	1.81	OR	7
2J0T	18600	71	2	55	59	198	0.0038	1.69	EI	1
2O3B	26875	69	16	135	29	7	0.0026	1.87	EI	1
2OUL	22568	93	94	18	10	1	0.0041	1.58	EI	1
2VDB	30672	75	145	260	380	183	0.0024	1.03	OX	8
4CPA	10526	56	22	43	48	14	0.0053	1.83	EI	1
**DimerBV5.0**	**NSRP**	**NIRP**	**SVM**	**RF**	**Logistics**	**Interaction logistic**	**P**	**Distance**	**category labels**	**category number**
2GAF	117448	129	662	52	8	525	0.0011	2.20	ER	3
2GTP	35910	59	42	125	15	6	0.0016	1.48	OG	6
2YVJ	36531	61	3	25	5	19	0.0017	1.90	ER	3
3A4S	11476	38	35	11	1	27	0.0033	2.72	EI	1
3AAD	37548	59	266	60	86	168	0.0016	2.14	OX	8
BAAD	37548	58	35	269	136	293	0.0015	1.45	OX	8
3FN1	16490	79	62	17	163	280	0.0048	1.24	ER	3
3K75	32109	54	189	2	72	300	0.0017	1.90	ER	3
3S9D	31408	69	69	441	127	42	0.0022	1.01	OR	7
3VLB	78764	96	18	604	19	11	0.0012	2.37	EI	1
4H03	138852	59	10	198	787	578	0.00043	2.62	ES	2
4IZ7	28014	44	622	213	297	1041	0.0016	1.74	EI	1
4M76	39772	43	74	153	434	303	0.0011	2.02	OR	7

**Figure 5:**
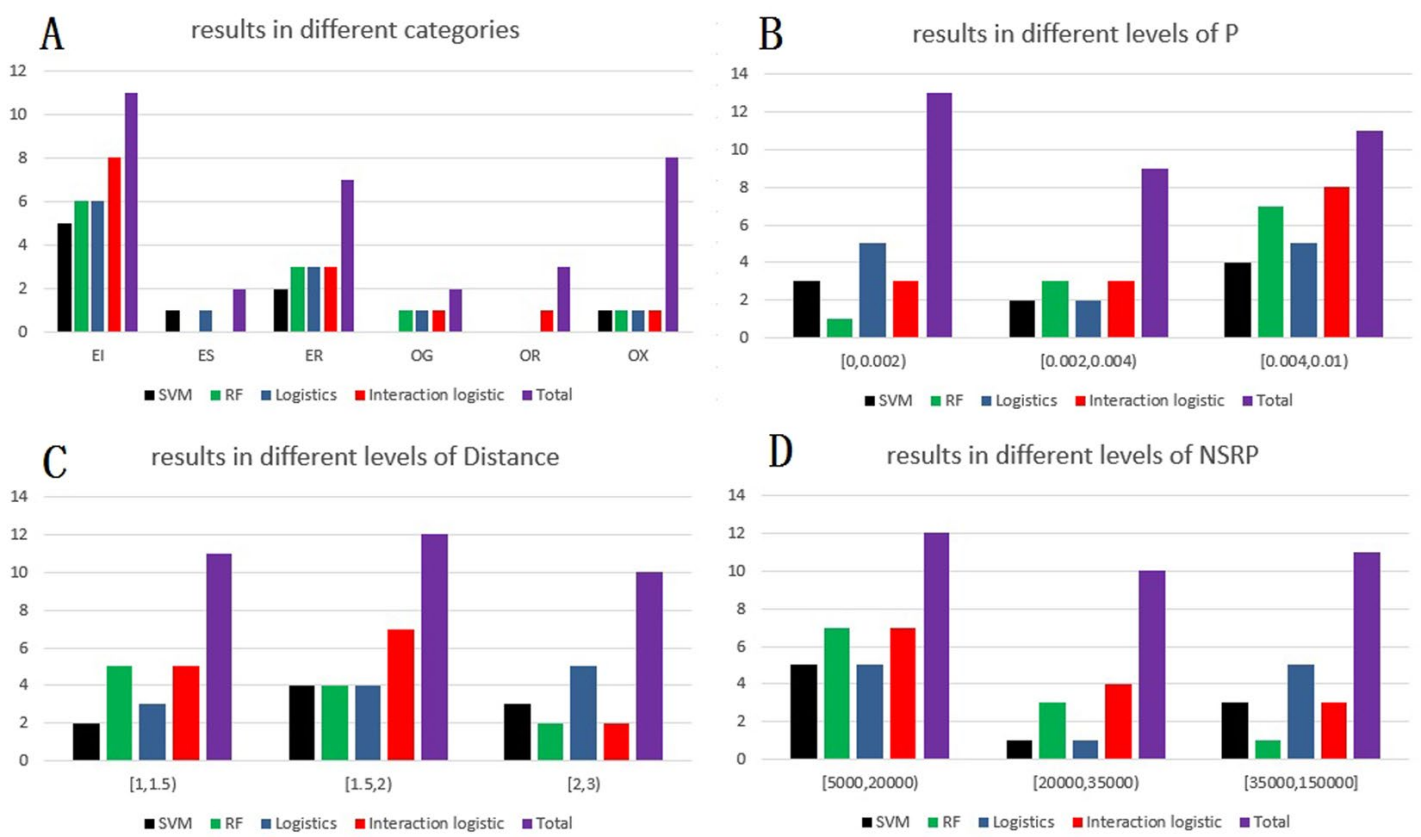
Histograms of four methods on BV 4.0 and 5.0 in different levels of attributes of dimers. The histograms recorded the numbers of correctly predicted dimers by choosing top 20 residue pairs in each dimer. (**A**) Histograms in different categories. (**B**) Histograms in different levels of P. (**C**) Histograms in different levels of Distance. (**D**) Histograms in different levels of NSRP.

In order to find the reasons of the above situation, we constructed Table 3 which included the analysis of variance (ANOVA) tables of four methods between first detected true positive ranking and dimer attributes. The ANOVA tables were computed according to Table S5 which contained the results of the linear models between first detected true positive ranking and dimer attributes. From Table 3, we could see that only category labels in the lasso logistic is significantly correlated with the first detected true positive ranking at significance level of 0.1. Besides, NSRP and P in SVM were statistical significant at significance level of 0.1. P in random forest and logistic regression with hierarchy interaction were significantly correlated with the first detected true positive ranking at significance level of 0.05. To visually present these visual thoughts, histograms of correctly predicted dimers were constructed by discretizing NSRP, P and Distance in to three parts in Fig. 5.

**Table 3:** ANOVA table between the first detected true positive ranking and protein attributes. Significant codes: 0 ‘***’ 0.001 ‘**’ 0.01 ‘*’ 0.05 ‘.’ 0.1 ‘ ’ 1.

Variables	SVM		RF		Logistic		Interaction logistic	
	F-value	Pr(>F)	F-value	Pr(>F)	F-value	Pr(>F)	F-value	Pr(>F)
Category	0.39	0.85	1.99	0.12	2.47	0.06.	0.49	0.78
P	3.74	0.07	4.55	0.04*	0.31	0.58	5.25	0.03*
Distance	0.06	0.81	0.26	0.62	1.09	0.31	0.3	0.59
NSRP	4.04	0.06	0.61	0.44	0.82	0.38	0.1	0.75

From Fig. 5, we could see more clearly the difference of the four methods in different values of attributes of dimers. When dimers belonged to EI, all methods performed well and logistic regression with hierarchy interaction performed best. When dimers belonged to OX, all method preform badly. When dimers belonged to ES, only SVM and logistic regression with lasso penalty had correct prediction and only logistic regression with hierarchy interaction had correct prediction when dimers were regarded to be OR. And we can saw that all methods performed well when the P of dimers were large. When the P of dimers were larger than 0.004, logistic regression with hierarchy interaction had the biggest number of correctly predicted dimers and when the P of dimers were smaller than 0.002, logistic regression with lasso penalty performed best. In addition, when the Distance of dimers were larger than 2, logistic regression with lasso penalty performed best. When the Distance of dimers were between 1.5 and 2, logistic regression with hierarchy interaction had the biggest number of correctly predicted dimers. Besides, when NSRP of dimers were larger than 35000, logistic regression with lasso penalty performed best. Random forest and logistic regression with hierarchy interaction performed pretty well when NSRP of dimers were smaller than 35000. And by comparing the histograms of different levels of NSRP and P, we found the histograms in different levels of P and NSRP were approximate inverse.

The result of Fig. 5B also proofed that the result of Table 3 that only P in lasso logistic was not significantly correlated with the first detected true positive ranking. But from Table 3, we couldn’t see the significant correlation between other protein attributes and the first detected true positive ranking expect category labels of lasso logistic and NSRP of SVM at significance level of 0.1. This result did not conform to the representation of Fig. 5. In order to find out the reason, we calculated the Pearson correlation coefficients between the attributes of proteins and constructed the analysis of variance table in Table S6. As a result, P was significantly correlated with Distance and NSRP. Distance was significantly correlated with NSRP. And the corresponding coefficient and p-value between P and Distance were −0.38 and 0.027. The coefficient and p-value between P and NSRP were −0.65 and 4.6e-5. The coefficient and p-value between Distance and NSRP were 0.54 and 0.0012. So P and NSRP has strong negative correlation and P and Distance has weak negative correlation. NSRP and Distance has positive correlation. And reflected in Fig. 5, it’s obviously that the performance between P and NSRP was opposite, while the performance of Distance was not obviously reverse compared to the performance of P and the performance of Distance was not the same as the performance of NSRP. But when Distance was large than 2, the prediction result was just like the result of NSRP that is large than 35000.

**Figure 6:**
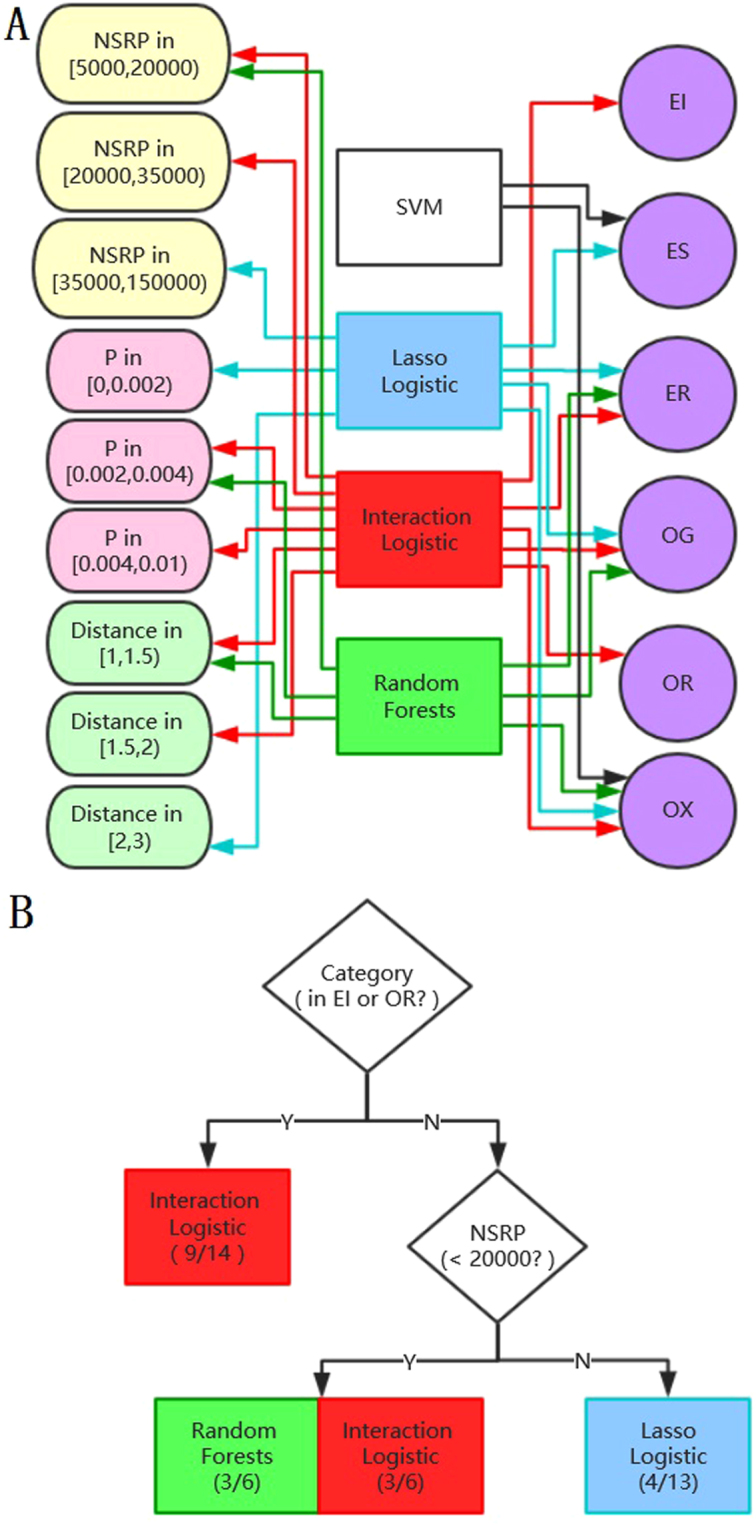
(**A**) Relationships between four methods and different level of attributes of dimers. (**B**) Reference methods in different level of attributes of dimers. The two numbers in parentheses under method represented the correct prediction number in corresponding group of dimers when choosing the number of selected residue-pairs in each dimer to be 20. For example, logistic regression with hierarchy interaction predicted 9 correct dimers in all 14 dimers belonging to EI and OR.

In Fig. 6A, we summarized the rules between different machine learning methods and different levels of attributes of dimers from Fig. 5 and above results. The relationship graph shown in Fig. 6A gave us an inspiration and a possible framework about how to choose suitable machine learning method by turning the arrows back. Although the relationships have not been mathematically and biological proven, but the phenomenon could not be denied and it sure could give us some guidance when treating with protein-protein data even other kind of protein data.

According to the above analysis, it is better to use lasso logistic when P was small because others methods performed worse as P decreased. But when facing a new dimer, we don’t know its interacting residue pairs so we cannot calculate its P and Distance. Luckily, P and Distance were strong correlated with NSPR which was known before prediction. Therefore, when NSRP was large which means P was small, using lasso logistic was a good choice. Besides, category labels also should be considered before prediction as it was not significantly correlated with P. As can be seen from Figs 5 and 6A, logistic regression with hierarchy interaction had biggest advantage when predicting the dimers belonging to EI and OR while random forest and lasso logistic predicted well when predicting the dimers belonging to ES. But we found that two dimers which belonged to ES all had large NSRP and small P. Thus we consider to choose logistic regression with hierarchy interaction when the new dimer belongs to EI or OR. When the new dimer doesn’t belong to EI or OR, take NSRP in to account. If NSRP of the new dimer is larger than 20000, lasso logistic should be chosen. Otherwise, choosing logistic regression with hierarchy interaction or random forest is the best choice. The above process was shown in Fig. 6B. In addition, different groups of dimers classified in different levels of attributes had a marked difference in prediction accuracy.

Compared with the single methods, the methods proposed in Fig. 6B were presented in Fig. 3 named “final 1” and “final 2”. The result of “final 1” was obtained by using random forest and the result of “final 2” was obtained by using logistic regression with hierarchy interaction when NSRP was less than 20000. It is important to note that we were most concerned with the prediction result choosing the number of selected residue-pairs in each dimer to be 20 as the conclusions in Figs 5 and 6 were all based on it. The vertical dotted line in Fig. 4 showed that the performances of “final 1” and “final 2” were both better than other four single methods. 16 correct dimers were found by “final 1” and “final 2” while the best of four single methods found 14 correct dimers. In addition, this phenomenon appeared not only when the number of selected residue-pairs in each dimer was 20. When the number of selected residue-pairs in each dimer was less than 20 or between 100 and 150, we could see that “final 2” was better than “final 1” and they were both better than other single methods. Similarly, “final 1” predicted more correct dimers than “final 2” and they both performed better than other four single methods when the number of selected residue-pairs in each dimer was between 20 and 100. So we could conclude that the methods proposed have advantage in prediction compared to four single methods if we select residue-pairs in each dimer less than 150.

In addition, for further studying the difference between different methods and understanding the meaning behind reference methods, we tried to learn variable selection of each method. As proposed methods in Fig. 6B didn’t include SVM, so the variable selection of SVM was ignored. Because random forest was applied without EasyEnsemble and feature engineering, it was easy to get its importance of variables in Table S7 using basis R code. And we found the most 5 important variables of random forest were the geometric features of receptor. The geometric features of ligand also made an important impact on prediction process in random forest. While the 8 hydrophilic features all had small importance in variable measurement. This result might tell us that for dimers whose NSRP are smaller than 20000, geometric features rather than hydrophilic features play an important role in prediction of protein-protein interactions. Logistic regression with lasso penalty and logistic regression with hierarchy interaction all used EasyEnsemble and feature engineering so we could get stable selection of variables by using stability selection algorithm. The details of stability selection were referred to Meinshausen, N. and Bühlmann, P.^23^ and our supplementary material. We chose main effects whose frequency were larger than 90% and chose interaction effects with frequency larger than 80%. The stability selection results of two methods were in Table S8. We could found that there were many of the same main effects and unlike random forest, there were many important hydrophilic features in models. And we found that eight interaction effects selected all contained at least one geometric feature. This phenomenon also matched the result of random forest and enhanced the inference that geometric features played a more important role than hydrophilic features in predicting dimers with NSRP less than 20000. Logistic regression with hierarchy interaction considered more representation of features so it performed well when predicting most protein-protein interactions. But facing dimers different with common sample, it became its weakness. And simplify of logistic regression with lasso penalty compared to logistic regression with hierarchy interaction meant that it was more robust when predicting distinctive dimers with NSRP larger than 20000.

## Discussion

Extremely imbalanced data have been studied for decades but many techniques used to overcome imbalance problem don’t have explicit mathematical properties. The main technique of this paper is EasyEnsemble and feature engineering. The prediction results of four methods using different algorithms in this paper indicate that this technique has a certain help in our prediction problems. But there is also a lack of theory and principals of this technique as four methods gain different improvement by using this technique. In this paper, we compared the effects of mean, median and weighted mean probabilities and finally chose the mean probabilities to assemble results of different classifiers as three ensemble methods performed similarly in our results. Ensemble method using mean treated all classifier fairly but might be affected by particularly poor or good results. Assigning weights to different classifiers might help improve the result of prediction but we have not found effective weighting method yet. The numbers of correct predicted dimers on training set were used as weights in our paper and its performance was not outstanding. Based on this, there is a lot of work can be done about how to generate, choose and assemble balanced samples optimally and how to expand variables reasonably in imbalance data problem.

This paper tended not to value the prediction effect of classification models or the specify algorithm. Instead, we compared four different classification methods on protein-protein to find the relationship between these methods and different classes of dimers. Global prediction results show that some dimers were easy to be predicted by all four methods while some dimers were only correctly predicted by some specified methods. The results suggest that different discriminants are suitable to recognize different protein-protein interface patterns. From this phenomenon, we then chose original properties of dimers including category labels and NSRP and constructed P and Distance to describe and distinguish the protein-protein data. Our final result was presented in Fig. 6. We found that different classification methods preferred different categories of dimers and different values of attributes of dimers. The relationship graph gave us a new idea that we could separate our dataset as we usually treated our dataset as a whole and chose one appropriate method to analyze it.

Specified machine learning method only generates discriminants with one or some linear or nonlinear formations. The common model used to recognize interface residue pairs can be regarded as a fixed linear or nonlinear function. It may be an appropriate way that interface residue pairs forming different interface patterns are recognized by different models as shown in Fig. 6A. Besides, although using methods presented in Fig. 6B had advantages compared to single methods, new protein-protein interactions data is still needed to validate the correctness of laws we found.

In our thought, different kinds of dimers may be corresponding to different distributions so it’s necessary to choose suitable methods. As shown in above results including ANOVA and variable selection, the choice between methods centered on logistic regression with lasso penalty and logistic regression with hierarchy interaction. It inspired us to construct that an algorithm that can detect the interaction in explanatory variables and response variable in unknown data. Besides, in protein-protein interaction problem, we are interested in the interaction position and the protein 3D structure. Next, more attributes of dimers such like 3D structure and more machine learning methods should be considered. More causal relationship and mathematical principles between methods and attributes of dimers need to be found and proved.

## Conclusions

In this paper, we used four effective classification methods to predict the interaction of residue pairs in dimers and compare their results. As we described the data of BV 3.0, BV 4.0 and BV 5.0, we found the distribution differs among different benchmark version, so it was important to deal with the data in difference problems. In addition, we combined EasyEnsemble and feature engineering to overcome the imbalance situation and learn deep information of protein-protein dataset. And this algorithm helped to enhance the effects of logistic models. Besides, this algorithm also could select stable variables and the selected variables are significant in logistic model.

Most important of all, we found some relationship between properties of proteins and different methods from the phenomenon that four methods behaved differently. We employed ANOVA and variable selection to study the reasons why different methods performed dissimilarly. And we constructed the relationship graph to give a guidance on the study of protein-protein interaction analysis and related problem. By controlling the amount of interacting residue pairs selected in a dimer under 150, our proposed methods performed better than all four single methods.

### Significance

It was found that different machine learning methods should be adopted for predicting different protein-protein interface patterns. This phenomenon may widely exist in different research areas, which reminds us that the scope of application of different methods should not be ignored when trying to solve a scientific problem.

## Electronic supplementary material

Supplementary Information

## References

[1] Braun P, Gingras AC (2012). History of protein–protein interactions: From egg–white to complex networks. Proteomics.

[2] Lin N, Wu B, Jansen R, Gerstein M, Zhao H (2004). Information assessment on predicting protein-protein interactions. BMC Bioinform..

[3] Chothia C, Janin J (1975). Principles of protein-protein recognition. Nature.

[4] koshland DE (1994). The Key-Lock Theory and The Induced Fit Theory. Angewandte Chemie-International Edition.

[5] Jones S, Thornton JM (1996). Principles of protein-protein interactions. Proc Natl Acad Sci USA.

[6] Esmaielbeiki, R., Krawczyk, K., Knapp, B., Nebel, J. C. & Deane, C. M. Progress and challenges in predicting protein interfaces. *Brief Bioinform* (2015). 10.1093/bib/bbv027 PMC4719070 25971595

[7] Maheshwari, S. & Brylinski, M. Predicting protein interface residues using easily accessible on-line resources. *Brief Bioinform* (2015). 10.1093/bib/bbv009 PMC6609008 25797794

[8] Xue LC, Dobbs D, Bonvin AM, Honavar V (2015). Computational prediction of protein interfaces: A review of data driven methods. FEBS Lett.

[9] Cortes C, Vapnik V (1995). Support-vector networks. Machine Learning.

[10] Breiman L (2001). Random Forests. Machine Learning.

[11] Hartigan JA, Wong MA (1979). Algorithm AS 136: A K-Means clustering algorithm. J. R. Stat. Soc. Ser. C Appl. Stat..

[12] Ester M, Kriegel HP, Sander J, Xu X (1996). A Density-Based Algorithm for Discovering Clusters in Large Spatial Databases with Noise. Kdd.

[13] Pirooznia M, Yang JY, Yang MQ, Deng Y (2008). A comparative study of different machine learning methods on microarray gene expression data. BMC genom..

[14] Williams N, Zander S, Armitage G (2006). A preliminary performance comparison of five machine learning algorithms for practical IP traffic flow classification. SIGCOMM Comput. Commun. Rev..

[15] Ahmad S, Mizuguchi K (2011). Partner-aware prediction of interacting residues in protein-protein complexes from sequence data. PLoS ONE.

[16] Bock JR, Gough DA, Bock JR, Gough DA (2001). Predicting protein–protein interactions from primary structure. Bioinformatics.

[17] Keskin O, Tuncbag N, Gursoy A (2016). Predicting Protein–Protein Interactions from the Molecular to the Proteome Level. Chem. Rev..

[18] Minhas FUAA, Geiss BJ, Benhur A (2014). PAIRpred: Partner-specific prediction of interacting residues from sequence and structure. Proteins.

[19] Šikić M, Tomić S, Vlahoviček K (2009). Prediction of protein–protein interaction sites in sequences and 3D structures by random forests. PLoS Comput. Biol..

[20] Ben-hur A, Ong CS, Sonnenburg S, Schölkopf B, Rätsch G (2008). Support Vector Machines and Kernels for Computational Biology. PLoS Comput. Biol..

[21] Koike A, Takagi T (2008). Prediction of protein–protein interaction sites using support vector machines. Protein Eng. Des. Sel..

[22] Chen XW, Liu M (2005). Prediction of protein–protein interactions using random decision forest *framework*. Bioinformatics.

[23] You ZH, Chan KCC, Hu P (2015). Predicting protein-protein interactions from primary protein *sequences* using a novel multi-scale local feature representation scheme and the random forest. PLoS ONE.

[24] Cox DR (1958). Corrigenda: The Regression Analysis of Binary Sequences. J. R.Stat. Soc. Series B Stat. Methodol..

[25] Lee H, Tu Z, Deng M, Sun F, Chen T (2006). Diffusion kernel-based logistic regression models for protein function prediction. Omics.

[26] Dhole K, Singh G, Pai PP, Mondal S (2014). Sequence-based prediction of protein-protein interaction sites with L1-logreg classifier. J. Theor. Biol..

[27] Lim M, Hastie T (2015). Learning interactions via hierarchical group-Lasso regularization. J. Comput. Graph. Stat..

[28] Qi Y, Bar-Joseph Z, Klein-Seetharaman J (2006). Evaluation of different biological data and computational classification methods for use in protein interaction prediction. Proteins.

[29] Vreven T (2015). Updates to the integrated protein-protein interaction benchmarks: Docking benchmark version 5 and affinity benchmark version 2. J. Mol. Biol..

[30] Liu TY (2009). Easyensemble and feature selection for imbalance data sets. IJCBS.

[31] Yin QY, Zhang JS, Zhang CX, Ji NN (2014). A novel selective ensemble algorithm for imbalanced data classification based on exploratory undersampling. Math. Probl. Eng..

[32] Meinshausen N, Bühlmann P (2010). Stability selection. J. R.Stat. Soc. Series B Stat. Methodol..

