# Supplementary material for: Different protein-protein interface patterns predicted by different machine learning methods
Source: Sci Rep. 2017 Nov 22;7:16023. doi: 10.1038/s41598-017-16397-z (PMC5700192; doi:10.1038/s41598-017-16397-z)
Supplement: Supplementary file 1 — Supplementary Information [file 41598_2017_16397_MOESM1_ESM.pdf]

Different protein-protein interface patterns predicted by different machine learning methods

Wei Wang[1], Yongxiao Yang[2], Jianxin Yin[1*] and Xinqi Gong[2*]
[1]Center for Applied Statistics and School of Statistics, [2]Mathematics Intelligence Application LAB, Institute for Mathematical Sciences, Renmin University of China, Beijing 100872, China.
*To whom correspondence should be addressed. E-mail: xinqigong@ruc.edu.cn；jyin@ruc.edu.cn

Supplementary Info

Data

The data that we used to predict and analyze interacting residue pairs are protein-protein docking benchmark dataset 5.0 (20), which contains 67 unbound state dimers. The benchmark dataset 5.0 was updated from benchmark dataset 3.0 and benchmark dataset 4.0. According to the version of benchmark, the data are separated to three subsets, whose details are presented in Table 1. In this paper, we tried to use the benchmark dataset 3.0 to train models and predict the interacting residue pairs in benchmark dataset 4.0 and 5.0. But different benchmark versions collect different kinds of dimers which may cause distribution difference, which will increase the difficulty of prediction but reduce the risk of over-fitting problem. As we can see from the Table S1, the percentages of interacting residue pairs differ in three datasets. Particularly, the percentage of interacting residue pairs of benchmark dataset 4.0 is two times as large as that of benchmark dataset 5.0. So it's reasonable to infer that the forecast result of benchmark dataset 4.0 would be better than that of benchmark dataset 5.0, which is confirmed by the actual results. The differences between datasets let us believe that it's necessary to research the different types of proteins.

Table S1. The basic information of dataset

BV [a]	ND [b]	NSRP [c]	NIRP [d]	P [e] (%)
3.0	34	1306311	2676	0.20
4.0 (updated)	20	556903	1436	0.26
5.0 (updated)	13	641870	848	0.13
Sum	67	2505084	4960	0.20

[a] BV: Benchmark Version; [b] ND: Number of Dimers; [c] NSRP: Number of Surface Residue Pairs; [d] NIRP: Number of Interacting Residue Pairs; [e] P: Percentage of interacting residue pairs.

Variables

We used 9 variables to describe each residue so there are totally 18 variables used to predict the

interaction. The target variable is set to a 0-1 variable named flag, flag=1 indicates that the residue pair interacts and flag=0 indicates that the residue pair does not interact. The specific explanations of the nine variables are shown in Table S2. The boxplots of 18 variables of receptor residue and ligand residues in all datasets are shown in Fig. S1. We can see from Fig. S1 that the distribution of receptor and ligand residues are similar. Interacting residue pairs have bigger absEA, relEA and IC and smaller EC, EV and H1 than non-interacting residue pairs in both receptor and ligand residue. H2 of receptor residues of interacting residue pairs is a bit smaller than that of non-interacting residue pairs. But H2 of ligand residues of interacting residue pairs is approximate to that of non-interacting residue pairs. The upper quartile of pK_a1 of interacting residue pairs is bigger than that of non-interacting residue pairs in both receptor and ligand residues. The upper quartile of pK_a2 of interacting residue pairs is bigger than that of non-interacting residue pairs in receptor residues but equal in ligand residues.

To compare the three datasets in details, we show the mean of 18 variables of the three datasets divided into interaction residue pairs and non-interaction residue pairs in Table S3. From Table S3, we can see more directly that the differences of the three datasets. For example, Benchmark dataset 5.0 have the biggest mean of absEA in the receptor residues of interacting residue pairs but have smallest mean of absEA in the ligand residues of interacting residue pairs. Similar but not the same patterns occur in relEA, EC, EV and IC. But the mean of H_1 of ligand residues of interacting pair-residues is bigger than that of non-interacting pair-residues in Benchmark dataset 4.0 while in Benchmark dataset 3.0 and 5.0, the situation is opposite. The same patterns happen in H2 of ligand residues and pK_a1 of receptor residues. It's an interesting fact that the mean of pK_a2 of ligand residues of interacting pair-residues is bigger than that of non-interacting pair-residues in the three datasets but in boxplot we cannot find their difference. These facts tell us that the three datasets may have different data distributions, so it's difficult to predict the updated data directly using early data. It's important to find the reasons that cause distribution difference. We try to find some regular patterns by constructing different kinds of models that predicting the interacting situation of pair-residues.

Table S2. The explanations of nine variables

Features	Abbreviation	Source
abs**olute** E**xterior solvent accessible** A**rea**	absEA	NACCES (21)
rel**ative** E**xterior solvent accessible** A**rea**	relEA	NACCES
E**xterior** C**ontact area with other residues**	EC	Qcontacts (22)
E**xterior** V**oid area**	EV	NACCES, Qcontacts
I**nterior** C**ontact area**	IC	Qcontacts
H**ydropathy index, version** 1	H1	Kyte, J. and Doolittle, R.F. (23)
H**ydropathy index, version** 2	H2	Eisenberg, D. (24)
pK_a1**: computation**	pK_a1	PROPKA3.1 (25)
pK_a2**: standard**	pK_a2	PROPKA3.1

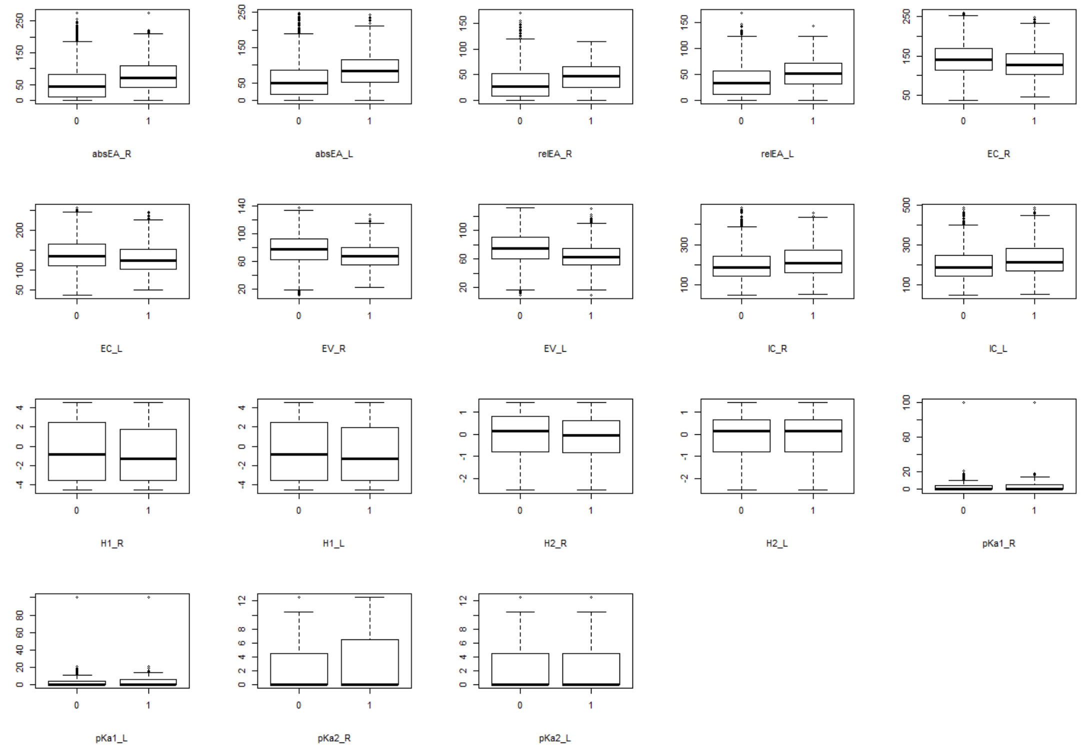

Fig. S1. Boxplots of 18 variables in all datasets. The right part shows the distribution of interacting residue pairs and the distribution of non-interacting residue pairs are shown in the left box.

Table S3. The mean of 18 variables between two interaction situation in three datasets

Receptor	BV 3.0 Flag=0	BV 3.0 Flag=1	BV 4.0 Flag=0	BV 4.0 Flag=1	BV 5.0 Flag=0	BV 5.0 Flag=1
absEA_R	51.355	76.538	50.726	70.808	51.724	85.557
relEA_R	32.737	47.159	32.273	43.713	32.641	50.098
EC_R	140.992	131.105	141.980	134.468	143.512	129.340
EV_R	77.461	67.154	77.013	69.432	77.253	65.084
IC_R	191.276	214.469	191.801	211.684	196.365	228.688
H1_R	-0.470	-0.940	-0.482	-1.027	-0.493	-0.954
H2_R	-0.022	-0.155	-0.037	-0.165	-0.050	-0.239
pK_a1_R	3.098	3.911	3.637	3.447	3.154	3.499
pK_a2_R	2.382	3.084	2.426	2.846	2.619	3.435
Ligand	BV 3.0 Flag=0	BV 3.0 Flag=1	BV 4.0 Flag=0	BV 4.0 Flag=1	BV 5.0 Flag=0	BV 5.0 Flag=1
absEA_L	55.456	84.579	60.865	92.407	52.671	79.828
relEA_L	35.545	50.175	38.697	54.980	33.863	49.057
EC_L	138.789	130.778	134.278	121.780	139.917	128.826
EV_L	75.156	65.207	73.484	61.951	76.775	65.512
IC_L	193.967	229.237	194.500	222.234	191.677	215.998
H1_L	-0.514	-0.903	-0.517	-0.431	-0.414	-0.829
H2_L	-0.042	-0.121	-0.044	-0.026	-0.003	-0.186
pK_a1_L	4.468	4.452	5.752	6.664	2.616	3.004

pK_a2_L	2.648	3.281	2.710	2.906	2.298	2.871

Tunings

The tunings of our models' basic parameter is described in this section.

Linear SVM model and random forest were trained using default parameters. Logistic model with lasso penalty chose optimal penalty parameter by 10-fold cross-validation. Parameters of logistic regression with hierarchy interaction including penalty, screen number and resampling times were gained by contrast experiments. Penalty controls the number of variables select in our models. Screen number means the number of main effects selected to interact with other effects in the model. Different kinds of logistic regression with hierarchy are trained on BV 3.0 and predicted on BV 4.0.

Fig. S2A was obtained by fixing resampling number to be 100, screen numbers to be 20 and varying penalty from 1/40 to 1/10 of smallest λ that choose none variables. From Fig. S2A, we find that the prediction result of gli10 is the worst and the results of gli20, gli30 and gli40 are close especially when abscissa is small. So we choose 1/20 of smallest λ that choose none variables as our penalty to ensure good prediction result and avoid over fitting due to too many variables selected in the model. By fixing resampling number to be 100, penalty to be 1/20 of smallest λ that choose none variables and varying screen number from 10 to 40, we got Fig. S2B. As we can see, the results of four choice of screen number are approached so we choose it to be 20 based on the same reason that we choose penalty. Fig. 3C contrast the results of different resampling times. In Fig. S2C, penalty is fixed to be 1/20 of smallest λ that choose none variables and screen number is fixed to be 20. From Fig. 3C, we find that when resampling number is 100, the prediction result is enough to match the result of 200 of resampling number so we choose resampling number to be 100. In the tuning process of parameters, we find that different parameters cause small change about the result which proves that logistic regression with hierarchy interaction have some robustness.

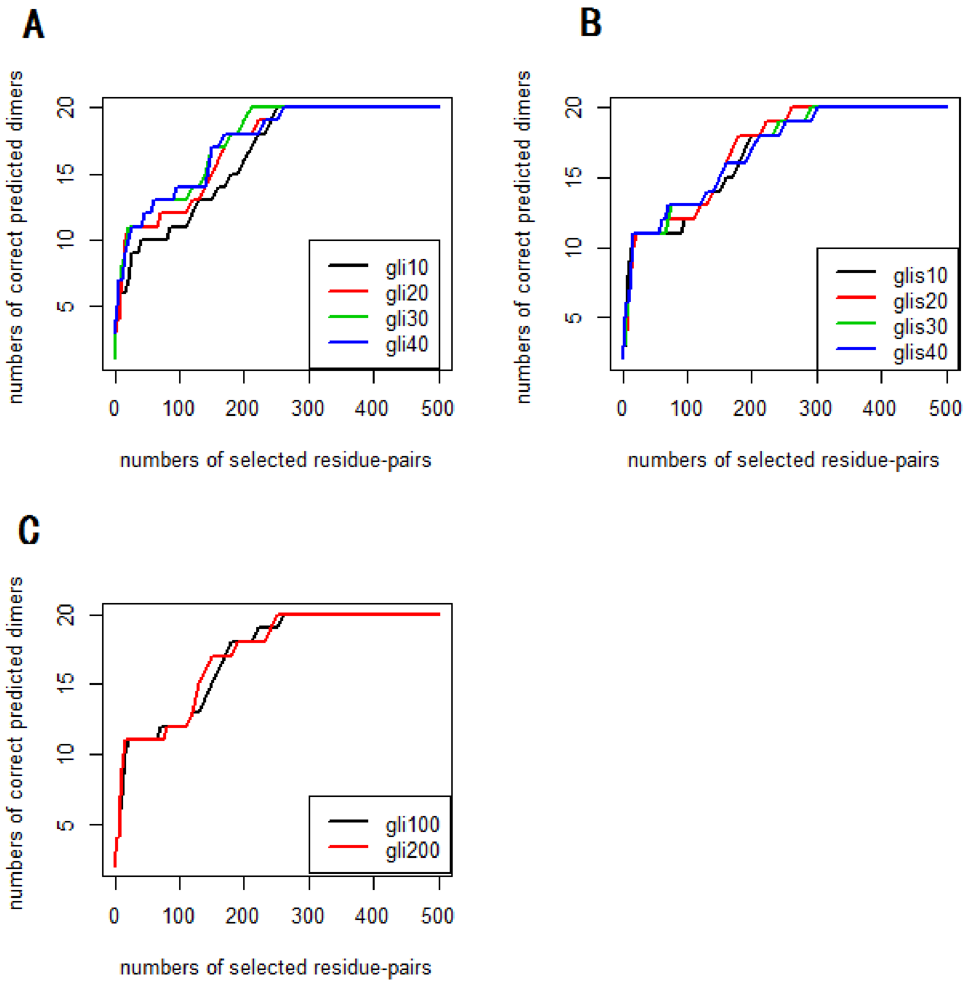

Fig. S2. Prediction results of logistic regression with hierarchy interaction on BV 4.0 using EasyEnsemble algorithm and feature engineering with different parameters. The abscissa means numbers of residue pairs chosen to be interacting pairs in a dimer and the ordinate means numbers of correct predicted dimers as long as there is one truly interacting residue pair chosen correctly. (A) Different lambda value. The result of gli10 represents 1/10 of smallest λ that choose none variables and so on. (B) Different screen numbers. Glis10 represents that screen number is 10 and so on. (C) Different resample numbers. Gli100 means that resample number is 100 and gli200 means that resample number is 200.

Differences between prediction of BV 4.0 and BV 5.0

We showed the forecast situations of top 20 residue pairs of four models using EasyEnsemble algorithm and feature engineering on BV 4.0 and BV 5.0 in Table 4 and Table 5. It's not unexpected that the outcomes of two dataset are inconsistent because we have already showed in data and variables section that three dataset may have different distribution and the percentage of interacting residue pairs differs up to a factor of two between BV 4.0 and BV 5.0. In Table 4, we see that logistic regression with hierarchy interaction perform best but in Table 5, it is logistic regression with lasso that has highest prediction accuracy. Especially we found that the accuracies of SVM and logistic regression with lasso have little difference between the prediction of BV 4.0

and BV 5.0 while the accuracies of random forest and logistic regression with hierarchy interaction reduce a lot from the prediction of BV 4.0 to BV 5.0. It told us that SVM and logistic regression with lasso may find more general patterns of protein-protein data while random forest and logistic regression with hierarchy interaction may find more details of data.

Let us observe the prediction results of four methods on BV 4.0 and BV 5.0 in more detail through Fig. S3. We can see that the logistic regression with hierarchy interaction performs not well when abscissa is not too small nor too large both on BV 4.0 and BV 5.0. Other three methods don't have this phenomenon in prediction process. It reminds us that if we want to choose a small amount of residue pairs to find interacting pairs, we could use logistic regression with hierarchy interaction. Besides, if a new dimer that is very different from train dataset needs to be predicted, SVM and logistic regression with lasso are good choices.

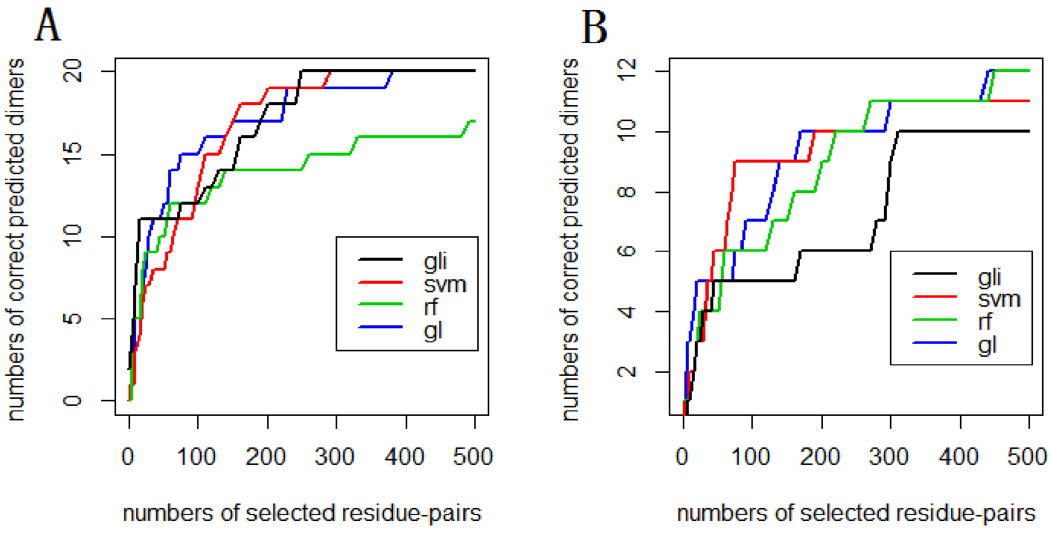

Fig. S3. Prediction results of four methods using different algorithms. The result of SVM was got only using EasyEnsemble. The result of random forest was got without EasyEnsemble and feature engineering. The results of logistic regression with lasso penalty, logistic regression with hierarchy interaction were obtained using both EasyEnsemble and feature engineering. The abscissa means numbers of residue pairs chosen to be interacting pairs in a dimer and the ordinate means numbers of correct predicted dimers as long as there is one truly interacting residue pair chosen correctly. (A) Prediction results on BV 4.0. (B) Prediction results on BV 5.0.

Stable variables selection

Random forest, logistic regression with lasso and logistic regression with hierarchy interaction can all choose significant variables during constructing the models. We collected all variables selection results in each resampling and select stable main effects. Stable variables of random forest were defined as the variables that fall in top 100 variables of importance in every resampling. Stable main effects of logistic regression with lasso and logistic regression with hierarchy interaction were defined as the main effects that are select by lasso equal or more than 80 times in a total of 100 times resampling. Comparing the stable variables selected by three methods, we found 13 variables that are all defined as their stable variables. These 13 variables

were absEA_R, relEA_R, EV_R, absEA_L, EV_L, IC_R, EV_R×EV_R, IC_R×IC_R, absEA_R/relEA_R, relEA_R/EV_R, EC_R/EV_R, pKa2_R/pKa2_L,IC_R/H2_R.

Using these stable variables we constructed a logistic regression on BV 3.0 and found these variables were very significant in the model. From the model showed in Table S4, we can get some obvious but interesting results. From the logistic model, we can know that if residue pairs have bigger absEA and IC of receptor residues, they are more likely to interact. If residue pairs have smaller EV of receptor and ligand residues, they are more likely to interact. At the meanwhile, larger square of EV of ligand residues and smaller square of IC of receptor residues may reduce the interacting probability. Besides, it’s interesting to see that the bigger the value of absEA of receptor residues divided resEA of receptor residues, the residue pairs are more likely to be non-interacting. And the smaller the value of pKa2 of receptor residues divided pKa2 of ligand residues, the residue pairs are more likely to interact. In addition, the larger the value of relEA of receptor residues divided EV of receptor residues, EC of receptor residues divided EV of receptor residues and IC of receptor residues divided H2 of receptor residues, the bigger the probability that residue pairs interact.

Table S4. Logistic model using stable selection variables

	Estimate	Std.Error	z value	Pr(>|z|)	
(Intercept)	-6.398	0.039	-164.638	0.000	***
absEA_R	0.268	0.046	5.888	0.000	***
relEA_R	0.053	0.066	0.795	0.427	
EV_R	-0.401	0.052	-7.738	0.000	***
absEA_L	0.064	0.052	1.229	0.219	
EV_L	-0.373	0.047	-7.872	0.000	***
IC_R	0.339	0.032	10.466	0.000	***
EV_R×EV_R	-0.222	0.020	-10.985	0.000	***
IC_R×IC_R	0.092	0.012	7.915	0.000	***
absEA_R/relEA_R	-0.056	0.020	-2.775	0.006	**
relEA_R/EV_R	0.061	0.014	4.460	0.000	***
EC_R/EV_R	0.026	0.009	3.035	0.002	**
pKa2_R/pKa2_L	-0.109	0.011	-9.855	0.000	***
IC_R/H2_R	0.051	0.007	7.478	0.000	***
